# Peopling of the North Circumpolar Region – Insights from Y Chromosome STR and SNP Typing of Greenlanders

**DOI:** 10.1371/journal.pone.0116573

**Published:** 2015-01-30

**Authors:** Jill Katharina Olofsson, Vania Pereira, Claus Børsting, Niels Morling

**Affiliations:** Section of Forensic Genetics, Department of Forensic Medicine, Faculty of Health and Medical Sciences, University of Copenhagen, Copenhagen, Denmark; St. Petersburg Pasteur Institute, RUSSIAN FEDERATION

## Abstract

The human population in Greenland is characterized by migration events of Paleo- and Neo-Eskimos, as well as admixture with Europeans. In this study, the Y-chromosomal variation in male Greenlanders was investigated in detail by typing 73 Y-chromosomal single nucleotide polymorphisms (Y-SNPs) and 17 Y-chromosomal short tandem repeats (Y-STRs). Approximately 40% of the analyzed Greenlandic Y chromosomes were of European origin (I-M170, R1a-M513 and R1b-M343). Y chromosomes of European origin were mainly found in individuals from the west and south coasts of Greenland, which is in agreement with the historic records of the geographic placements of European settlements in Greenland. Two Inuit Y-chromosomal lineages, Q-M3 (xM19, M194, L663, SA01 and L766) and Q-NWT01 (xM265) were found in 23% and 31% of the male Greenlanders, respectively. The time to the most recent common ancestor (TMRCA) of the Q-M3 lineage of the Greenlanders was estimated to be between 4,400 and 10,900 years ago (y. a.) using two different methods. This is in agreement with the theory that the North Circumpolar Region was populated via a second expansion of humans in the North American continent. The TMRCA of the Q-NWT01 (xM265) lineage in Greenland was estimated to be between 7,000 and 14,300 y. a. using two different methods, which is older than the previously reported TMRCA of this lineage in other Inuit populations. Our results indicate that Inuit individuals carrying the Q-NWT01 (xM265) lineage may have their origin in the northeastern parts of North America and could be descendants of the Dorset culture. This in turn points to the possibility that the current Inuit population in Greenland is comprised of individuals of both Thule and Dorset descent.

## Introduction

Information on the genetic diversity and structure of human populations is highly relevant in many fields of genetics. It is, for example, very important to control for population stratification in genetic association studies to exclude false-positive associations due to population specific characteristics [1]. Furthermore, in forensic genetics, information on genetic diversity is vital for proper evaluation of the weight of the evidence [2]. Analyses of genetic markers in the non-recombining regions of the human Y chromosome have proven to be highly informative for population genetic studies of human migrations, for example reviewed by Oppenheimer [3].

The North Circumpolar Region has been colonized by humans for the last 5,000 years. The early Paleo-Eskimos began to expand from Beringia along the northern coastlines of Alaska and Canada approximately 4,500 years ago (y. a.), and the Saqqaq Paleo-Eskimos reached Greenland approximately 4,000–4,500 y. a. [4,5]. Approximately, 3,000–3,500 y. a., the Dorset culture developed in Eastern Canada around Hudson Bay and spread towards the west and north, reaching Greenland approximately 2,800 y. a. and completely replacing the previous Paleo-Eskimo cultures [4,5]. Approximately 1,000 y. a., the Thule culture emerged along the North Alaskan coast [5]. They then spread quickly throughout the North Circumpolar Region and reached Greenland approximately 800–1,000 y. a. [4,5]. Controversy still exists regarding the extent of admixture between the Inuit populations of the Dorset and Thule cultures. There is evidence that the Dorset culture was extinct prior to the Thule expansion and that the current North Circumpolar populations stem entirely from the Thule [6]. However, other studies argue that the Dorset and Thule cultures coexisted for at least 200 years, which would have allowed interbreeding [7,8].

The first European settlement in Greenland, by the Norse from Norway and Iceland, is dated to 985 AD [9]. The Norse primarily settled along the west and south coasts of Greenland [9,10]. However, it is thought that the Norse population did not survive, and after 1450 AD, there is no evidence of any Norse settlements in Greenland [9,10]. In 1721 AD, Danish and Norwegian settlers arrived in Greenland [9,11]. The Scandinavians established trading colonies that essentially correspond to the major communities found along the coastline of Greenland [9,11].

In accordance with the historical records, the contemporary Greenlandic population is admixed containing an Inuit component and a European component [9,12,13]. Studies utilizing mitochondrial DNA (mtDNA) have shown that the vast majority of the maternal lineages of Greenlanders are of Inuit origin [5]. In contrast, studies of the Y chromosome have shown that the male population is highly admixed, with a European component accounting for up to 45% of the Y-chromosomal lineages of Greenlanders [9,13]. Approximately 50% of the male Greenlandic population belong to the paragroup P*-M45 (xM19, M173) [13]. These individuals are hypothesized to belong to the Y-chromosomal SNP haplogroup (Y-HG) Q-M242 and a preliminary study of 167 male individuals from Greenland supports this hypothesis [14].

As a result of the non-recombinant nature of the human Y chromosome, all human Y chromosomes can be assigned to a Y-chromosomal lineage. The Q-M242 Y-chromosomal lineage is considered to be a major paternal lineage among Native American populations [15–19], although sub-haplogroups of Q-M242 are found throughout the world at low frequencies [19]. The main branches within the Q-M242 haplogroup discussed in this study are shown in Figure A in S1 File. In this paper, Q-M242 is used to define all of the individuals with a derived allele at the M242 locus, whereas Q*-M242 defines the individuals with a derived allele at M242 and ancestral alleles at all of the typed downstream-markers within the Q-M242 lineage. The same logic is used for all of the other lineages and sub-lineages mentioned throughout this study.

The aim of this study was to perform a detailed investigation of the distributions of European and Inuit Y-HGs in the contemporary male population of Greenland. A total of 73 Y-chromosomal single nucleotide polymorphisms (Y-SNPs) were studied. To refine the male population of Greenland, 17 Y-chromosomal short tandem repeats (Y-STRs) were also investigated. Particular attention was given to the Inuit component of the population in Greenland to gain knowledge regarding the North Circumpolar Inuit groups. In addition, the European Y chromosomes in the male Greenlandic population were defined and compared to those of the Danish population.

The results showed that 40% of the male Greenlandic population carries Y chromosomes of European origin. The European lineages were primarily found in south and west Greenland. In regards to the Inuit component, our results points to a gene flow between the Inuit populations of the Dorset and Thule cultures, which can help to elucidate the current debate on the Inuit founders of the Greenlandic population.

## Results

### The Y-chromosomal haplogroup diversity and distribution in Greenlanders

Overall, 21 Y-HGs were found in the male Greenlandic population (S1 and S2 Table). The samples were grouped based on the place of birth of the individuals as outlined in the Materials and Methods (see Table 1 and Figure B in S1 File). Two Y-HG Q-M242 lineages were found to be prominent in all five regions of Greenland: Q-NWT01 (xM265) (31%) and Q-M3 (xM19, M194, L663, SA01 and L766) (23%). Approximately 40% of the male Greenlanders belong to one of the three major European Y-HGs: I-M170 (14%), R1a-M513 (7%) and R1b-M343 (19%). European Y-chromosomal lineages were predominately found in South and West Greenland (Table 1), and the vast majority of the Danish individuals belonged to the Y-HGs I-M170 (39%), R1a-M513 (17%), or R1b-M343 (37%) (S1 and S2 Table).

**Table 1:** The distribution of the Y-chromosomal haplogroups in Greenlanders and in the five sub-populations in Greenland.

**Y-chromosomal haplogroup**	**East Sermersooq (N = 68)**	**Kujalleq (N = 24)**	**West Sermersooq (N = 64)**	**Qeqqata (N = 27)**	**Qaasuitsup (N = 44)**	**Greenland (N = 227)**
Q-NWT01 (xM265)	37 (54%)	4 (17%)	10 (16%)	5 (19%)	14 (32%)	70 (31%)
Q-M3 (xM19, M194, L663, SA01 and L766)	19 (28%)	10 (42%)	11 (17%)	3 (11%)	9 (20%)	52 (23%)
I-M170	2 (3%)	2 (8%)	14 (22%)	3 (11%)	11 (25%)	32 (14%)
R1a-M513	0 (0%)	2 (8%)	8 (13%)	4 (15%)	3 (7%)	17 (7%)
R1b-M343	7 (10%)	6 (25%)	19 (30%)	9 (33%)	4 (9%)	45 (20%)
Other	3 (4%)	0 (0%)	2 (3%)	3 (11%)	3 (7%)	11 (5%)

A total of 11 (4.6%) Greenlandic and 13 (5.4%) Danish Y chromosomes could not be defined by the five multiplexes used in this study. Therefore, Y-STR profiles were used to predict the Y-HG of these 24 Y chromosomes (S2 Table). A single Greenlandic Y chromosome was predicted to belong to Q-M242. However, this chromosome did not bear the derived allele at the M242 locus in this study or in a previous study [13].

The overall diversity of the Y-HGs in Greenland was 0.84 (Table A in S1 File). The highest diversity (0.92) was found in West Sermersooq and the lowest (0.63) was in East Sermersooq (Table A in S1 File). Using pairwise genetic distances (*F*_ST_), East Sermersooq was found to be significantly different (p<0.05) from the Kujalleq, West Sermersooq and Qeqqata populations (Table B in S1 File).

The Greenlandic Y chromosomes were divided into three groups: (1) Inuit (Q-NWT01 (xM265) and Q-M3 (xM19, M194, L663, SA01 and L766)), (2) European (I-M170, R1a-M513 and R1b-M343) and (3) ‘other’ (Y chromosomes that were undefined in this study). The distribution of these three groups in the five geographic regions of Greenland can be seen in Figure B in S1 File. There was a decrease in the frequency of the Inuit Y-HGs along the coastline, from 82% in East Sermersooq to 30% in Qeqqata in the West (Fisher’s exact test: p<0.001 between East Sermersooq and Qeqqata) (Table 1). A corresponding increase in the European Y-HGs was identified as well, from 13% in East Sermersooq to 64% in West Sermersooq and 59% in Qeqqata. The municipally of Qaasuitsup did not fit this pattern. Qaasuitsup consists of multiple small communities spread over a large geographic area with only a few individuals in the dataset from each community. More data would be needed to properly characterize the frequencies of the Y-HGs in Qaasuitsup.

### The Y-chromosomal haplotype diversity of Greenlanders

**Figure 1:**
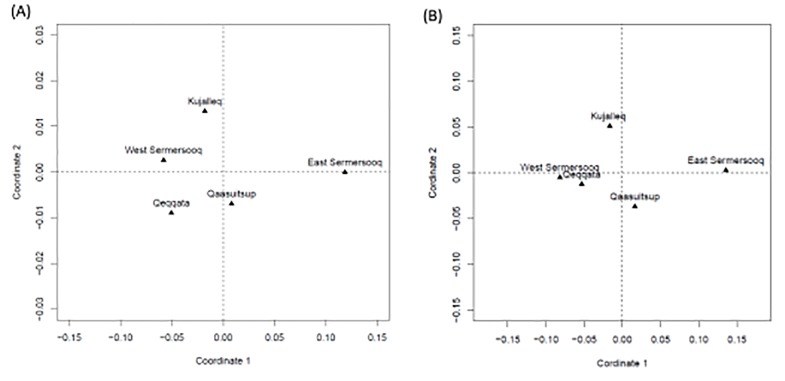
The multidimensional scaling analysis of the pairwise genetic distances between the five sub-populations in Greenland calculated for 15 Y-STRs. (a) *R*_ST_, (b) Discrete Laplace method.

**Intra-population comparisons**. The Y-chromosomal variation in Greenlanders was further refined by Y-STR analysis. The overall Y-STR diversity in the Greenlanders was 0.98 (Table C in S1 File). As was observed for the Y-SNPs, the highest Y-STR haplotype diversity (0.99) was found in individuals from West Sermersooq and the lowest (0.91) was found in individuals from East Sermersooq (Table C in S1 File). The pairwise genetic distances (*R*_ST_) and the genetic distances based on the discrete Laplace method [20] were calculated and the two methods for representing genetic distances produced similar multidimensional scaling (MDS) plots (Fig. 1). The population of East Sermersooq was distant from the four other populations in Greenland. Using the *R*_ST_, the population of East Sermersooq was found to be significantly different (p<0.05) from all of the other populations in Greenland (Table D in S1 File). On the converse, using the discrete Laplace method, the population of East Sermersooq was not significantly different from that of Kujalleq (Table E in S1 File).

**Figure 2:**
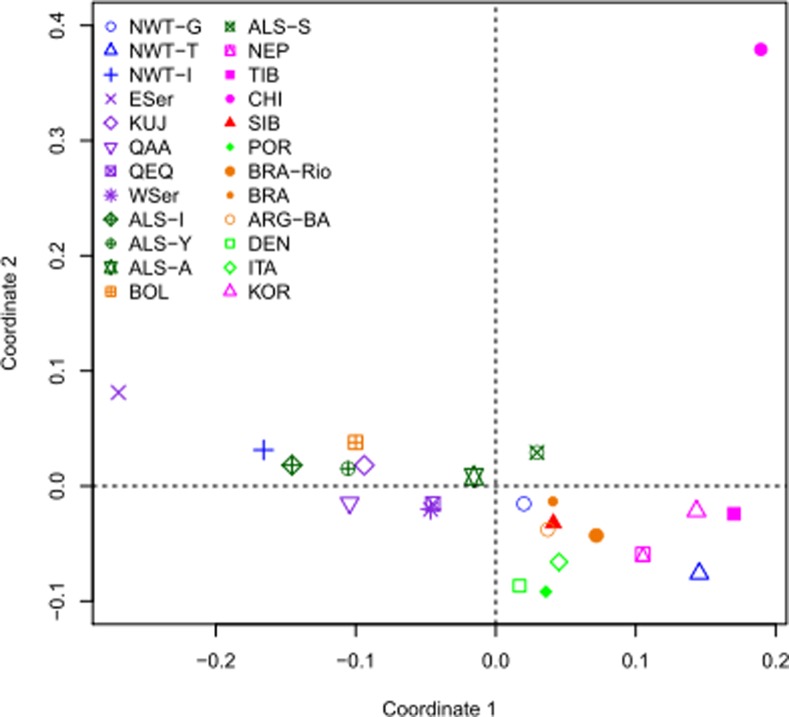
The multidimensional scaling analysis based on the pairwise *R*_ST_ genetic distances calculated for 15 Y-STRs. Population codes: NWT-G – Canadian North Territories (Gwich’in); NWT-T – Canadian North Territories (Tlicho); NWT-I – Canadian North Territories (Inuvialuit); ESer – East Sermersooq; KUJ – Kujalleq; QAA – Qaasuitsup; QEQ – Qeqqat; WSer – West Sermersooq; ALS-I – Alaska (Inupiat); ALS-Y – Alaska (Yupik); ALS-A – Alaska (Athabaskan); BOL – Bolivia (Multiethnic); ALS-S – Alaska (Southeastern); NEP – Nepal (Tamang, Newr, and Kathmandu); TIB – Tibet; CHI – Han Chinese; SIB – Siberia (Altai-kizhi, Chelkans, Kumandins, and Tubalars); POR – Portugal; BRA-Rio – Brazil (Rio); BRA – Brazil; ARG-BA – Argentina (Buenos Aires); DEN – Denmark; ITA – Italy; KOR – South Korea.

**Inter-population comparisons**. The inter-population variations were estimated using pairwise genetic distances (*R*_ST_) and visualized in an MDS plot (Fig. 2). Data from relevant populations for comparisons were included in the analyses (see Table F in S1 File for references). The population of East Sermersooq did not group with the other Greenlandic populations but was found to be closest to the Inuvialuit population of the Canadian Northwest Territories (Fig. 2). With the exception of East Sermersooq, the Greenlandic populations were found to be closer to the Alaskan populations than to the other populations (Fig. 2). The populations of Qeqqata and West Sermersooq were closer to the European populations than the other sub-populations in Greenland (Fig. 2).

### The diversity and relative chronology of the Y-chromosomal lineages of Greenlanders

The Y-STR diversities and mean pairwise differences (MPD) within the Y-HGs of Greenlanders are presented in Table 2. For comparison, the same parameters were estimated in 162 male Danes from [21] (Table 2). The Y-STR diversities of the Y-HGs I-M170 and R1b-M343 in the Greenlanders were similar those of the Danes (Table 2). However, the diversity of the R1a-M513 chromosomes in Greenlanders was smaller than that of the Y-HG in the Danes.

**Table 2:** Estimates of the diversities of the (1) Inuit (QNWT01 (xM265) and Q-M3 (xM19, M194, L663, SA01 and L766)) and (2) European (I-M170, R1a-M513 and R1b-M343) Y-chromosomal lineages.

**Haplogroup**	**Population**	**N**	**h**	**HT diversity**	**MPD**	**Vp**
Q-NWT01 (xM265)	Greenlanders	70	22	0.901 +/− 0.020	3.08 +/− 1.62	0.27
Q-M3 (xM19, M194, L663, SA01 and L766)	Greenlanders	52	17	0.856 +/− 0.031	2.50 +/− 1.37	0.09
I-M170	Greenlanders	32	26	0.986 +/− 0.012	5.48 +/− 2.71	0.35
I-M170	Danes	69	62	0.996 +/− 0.004	5.93 +/− 2.86	0.40
R1a-M513	Greenlanders	16	9	0.767 +/− 0.113	3.05 +/− 1.68	0.13
R1a-M513	Danes	29	28	0.998 +/− 0.010	5.88 +/− 2.89	0.31
R1b-M343	Greenlanders	45	36	0.980 +/− 0.013	5.93 +/− 2.89	0.39
R1b-M343	Danes	64	64	1.000 +/− 0.003	5.76 +/− 2.79	0.34

N: Number of samples; h: Number of haplotypes; HT: Haplotype; MPD: Mean pairwise difference; Vp: Intra-population variance.

The Inuit Y-chromosomal lineages in the Greenlanders, HG Q-NWT01 (xM265) and Q-M3 (xM19, M194, L663, SA01 and L766), had approximately half of the Y-STR diversity compared to the European lineages R1b-M343 and I-M170 (Table 2). Although the lower diversity can be caused by other factors such as smaller effective population size, patterns of male dispersal or selection against deleterious alleles, the lower diversities in the two Q-M242 lineages may indicate that they arose more recently than the European lineages. The lowest Y-STR diversity was observed in the Q-M3 (xM19, M194, L663, SA01 and L766) lineage, indicating a more recent origin than the Q-NWT01 (xM265) lineage.

**Figure 3:**
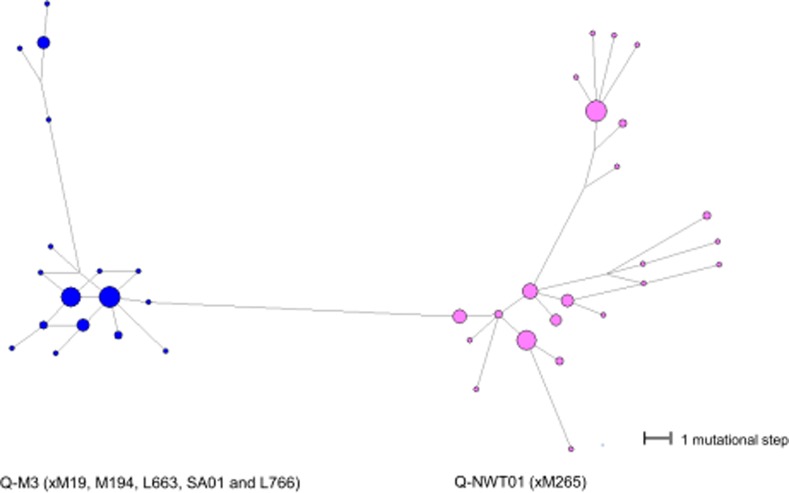
The median joining network based on the Y-STR haplotypes of the two Inuit Y chromosome lineages, Q-NWT01 (xM265) and Q-M3 (xM19, M194, L663, SA01 and L766) in Greenlanders. The circle sizes indicate the number of individuals with shared Y-STR haplotypes. The smallest circles represent one individual. The lengths of the connecting branches indicate the number of mutational steps. The shortest branches represent one mutational step.

All of the Y-STR haplotypes belonging to Q-M242 were connected in a median joining network (Fig. 3). The Inuit Y-chromosomal lineages formed two distinct clusters in the network. The majority of the individuals in the Q-M3 (xM19, M194, L663, SA01 and L766) cluster belonged to one tight group with short branches between the haplotypes. A smaller group of individuals formed a more distant sub-cluster within the Q-M3 (xM19, M194, L663, SA01 and L766) cluster. Conversely, the individuals belonging to the Y-HG Q-NWT01 (xM265) formed a less defined network with longer branches between the haplotypes (Fig. 3). This is a reflection of the larger MPD between haplotypes within Q-NWT01 (xM265) compared to the MPD between the haplotypes within Q-M3 (xM19, M194, L663, SA01 and L766) (Table 2).

**Figure 4:**
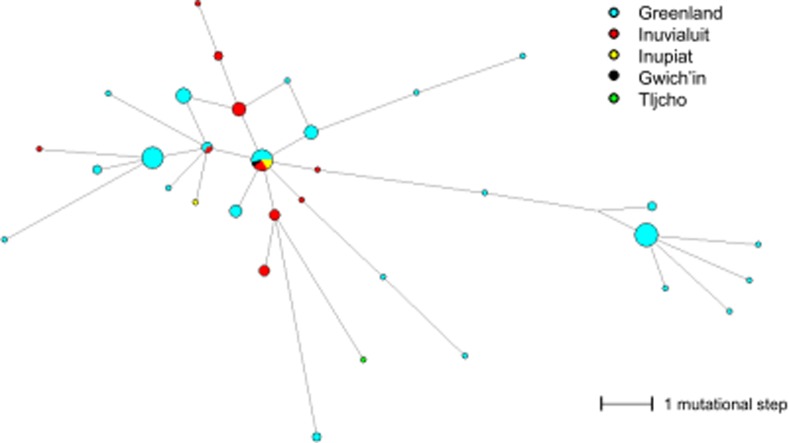
The median joining network of the individuals with Y-HG Q-NWT01 (xM265). The data from this study and from the study by Dulik et al [13] were combined. The circle sizes indicate the number of individuals with shared Y-STR haplotypes. The smallest circles represent one individual. The lengths of the connecting branches indicate the number of mutational steps. The shortest branches represent one mutational step.

The Y-STR haplotypes of the Q-NWT01 (xM265) chromosomes from this study and from Dulik et al. [22] were connected through a median joining network (Fig. 4). There were few shared Y-STR haplotypes between the Greenlandic population and the Inuit population from the Canadian Northwest Territories. However, the apparent founder haplotype was shared, indicating a common origin.

To further compare the relative chronology between Q-NWT01 (xM265) and Q-M3 (xM19, M194, L663, SA01 and L766) in the Greenlanders, the time to the most recent common ancestor (TMRCA) was estimated for the two haplogroups. The TMRCA for the Q-NWT01 chromosomes using ρ-statistics indicated a coalescent event that occurred approximately 7,000 y. a. (Table 3). Similarly, a coalescent event for the Q-M3 (xM19, M194, L663, SA01 and L766) lineage in the Greenlanders was estimated to have occurred approximately 4,400 y. a. using ρ-statistics [23]. The estimates of the TMCRA using the Bayesian approach implemented in the software program Batwing were larger than the estimates using ρ-statistics, approximately 14,300 and 10,900 y. a. for Q-NWT01 (xM265) and Q-M3(xM19, M194, L663, SA01 and L766) respectively (Table 3).

**Table 3:** The estimated time to the most recent common ancestor (TMRCA) for the Y-HGs Q-NWT01 (xM265) and Q-M3 (xM19, M194, L663, SA01 and L766) in the Greenlandic population.

**Haplogroup**	**N**	**ρ-statistics TMRCA (y. a.)**	**ρ +/− σ**	**Batwing TMRCA (y. a.)**	**Batwing 95% CI**
Q-NWT01 (xM265)	70	7,038 +/− 1,945	2.91 +/− 0.81	14,318	7,185–31,509
Q-M3 (xM19, M194, L663, SA01 and L766)	52	4,366 +/− 1,274	1.81 +/− 0.53	10,921	5,347–22,620

N: Sample size; TMRCA: Time since most recent common ancestor; y.a: Years ago; CI: Confidence interval (years).

## Discussion

In accordance with previous studies [13,14], we found that the contemporary male population in Greenland is admixed, with Inuit Y-chromosomal lineages accounting for approximately 50% of the Y chromosomes investigated. The male Greenlandic population was divided into five sub-populations based on geographic region of birth. The sub-population of West Sermersooq was the most diverse. The geographic region of West Sermersooq includes the capital city, Nuuk, and was therefore expected to show the greatest genetic diversity. On the converse, the region of East Sermersooq had the lowest genetic diversity. East Sermersooq is comprised of two remote communities (Figure B in S1 File); therefore it was not surprising that the genetic diversity was low in this region. The calculations of the pairwise genetic distances indicated that the population of East Sermersooq is different from the other populations in Greenland. A similar structure within the Greenlandic population was previously reported using autosomal and X-chromosomal markers [12]. Furthermore, with the exception of the Inuvialuit population of the Canadian Northwest Territories, the population from East Sermersooq differed from the other populations from different regions of the world that were included in the comparative analysis. Similar to the population from East Sermersooq, the Inuvialuit population had a high frequency of Q-NWT01 (xM265) [22], therefore, it is not surprising that the two populations did not differ from each other.

Approximately 40% of the Y-HGs in the male Greenlandic population were found to be of European origin. Only considering the European Y-HGs (I-M170, R1a-M513 and R1b-M232) in Greenland, the relative frequencies of these Y-HGs in the Greenlanders resembled those observed in the male Danish population examined in this study and other male Scandinavian [24–26] and Icelandic populations [27]. However, as previously reported [14], the distribution of the European Y-HGs in the Greenlanders is not uniform. Along the south and west coasts of Greenland, the European Y-HG lineages were found at a higher frequency (40–60%). This corresponds well with the historic records of the geographic locations of the European settlements created by the Norse during the period from 1000–1200 AD, and later by Scandinavians in the 18th century [9].

The Y-STR haplotype diversities and the MPDs within the European Y-chromosomal lineages I-M170 and R1b-M343 in the Greenlanders were similar to each other and to the variations found in the same Y-HGs in the Danes. This can, for example, be explained by continuous gene flow from Scandinavia, primarily from Denmark. However, multiple founders of the European lineages in Greenland can also explain this observation. Some of the diversity could, for example, be attributed to the multiple settlements in Greenland by Europeans. Previous studies have tried to differentiate between gene flow from the Norse and from the Scandinavians [9]. However, no conclusions could be drawn [9]. In strong contrast to the results of this study and previous studies [9,13], typing of the mtDNA in the Greenlandic population shows an almost complete fixation of Inuit maternal lineages [5]. The European gene flow detected in Greenlanders can therefore primarily be attributed to males.

Two Y-chromosomal lineages, Q-M3 (xM19, M194, L663, SA01 and L766) and Q-NWT01 (xM265) were found to be prominent in Greenlanders. The Q-M3 lineage has previously been found among Native American populations throughout North and South America [18,28,29], whereas Q-NWT01 (xM265) was previously reported at high frequency in an Inuit population of the Canadian Northwest Territories [22].

The Y-chromosomal lineages Q-MEH2 (xL54) and Q-L54 (Figure A in S1 File) are associated with the initial migration into North America [30]. Soon after the initial entry the derived M3 variant is hypothesized to have appeared on a Q-L54 chromosome [18,30]. Humans carrying this derived state at M3 quickly spread across North and South America [18,30]. Both Q-M3 and Q-MEH2 were previously identified in Inuit populations [22,31–34]. Individuals belonging to Q-MEH2 can theoretically be carriers of the derived state at NWT01 (Figure A in S1 File).

In Greenlanders, the Q-NWT01 (xM265) and Q-M3 (xM19, M194, L663, SA01 and L766) lineages clustered to different regions of the island (Table 2), which may indicate that the current male Inuit population in Greenland is comprised of individuals who are descendants from two different populations. However, a single source population with at least two founding Y-chromosomal Q-M242 lineages can also explain these findings. The observed genetic distribution in Greenlanders can, for example, be explained by separate migration patterns of the two Q-M242 lineages within Greenland after the initial migration onto the island. To further investigate the subject, the relative chronology and diversity of the two Inuit Y-chromosomal lineages were investigated in more detail, and compared to a recent study of Inuit populations from the Canadian Northwest Territories [22].

The estimated TMRCA (4,400–11,000 y. a.) of the Q-M3 (xM19, M194, L663, SA01 and L766) in Greenland was lower than that of the Q-NWT01 (xM265) (7,000–14,300 y. a.). This indicates that in Greenland, the Q-M3 (xM19, M194, L663, SA01 and L766) lineage has a more recent origin than the Q-NWT01 (xM265) lineage. However, the estimates are largely overlapping; therefore, caution should be taken when making comparative conclusions.

The estimated TMRCA of the Q-M3 (xM19, M194, L663, SA01 and L766) lineage in Greenlanders is lower than that estimated for the same lineage among the Inuit populations of the Canadian Northwest Territories [22]. The same was observed for the haplotype diversity and the MPD [22]. These observations are consistent with the theory that the derived M3 variant appeared in Beringia or Alaska and that individuals carrying the derived stated at M3 migrated towards the East across North American and eventually reached Greenland [4,5]. It is likely that individuals carrying the derived state at M3 are descendants of the Thule culture, which has its origin in North Alaska.

The estimated TMRCA of the Q-NWT01 (xM265) lineages in Greenland (7,000–14,300 y. a.) was approximately three-times older than that of the same lineage in the Canadian Northwest Territories, which was 2,900–5,700 y. a [22]. However, the 95% confidence intervals of the Batwing estimates of the TMCRA are overlapping; therefore, the difference is not statistically significant. The Y-STR haplotype diversities were comparable for the Q-NWT01 (xM265) lineage in the two populations. However the MPD between the haplotypes within the Q-NWT01 (xM265) lineage was larger for the Greenlandic population than for the Inuvialuit population of the Canadian Northwest Territories. All of the individuals belonging to Y-HG Q-NWT01 (xM265) appeared to share a common origin (Fig. 4). The low number of shared Y-STR haplotypes between the populations is most likely a result of founder effects and/or bottlenecks and reflects the effects of genetic drift on Y-STRs in the small isolated communities across the North Circumpolar Region.

The presence of Y-HG Q-NWT01 (xM265) (as well as Y-HG C-P39 which was not studied in this work) in the Inuit populations was previously suggested to represent a second expansion of the human population within North America via a migration from the Northwest region [22]. However, the older TMRCA of the Q-NWT01 (xM265) lineage in Greenland than in the Canadian Northwest Territories could indicate that the Q-NWT01 (xM265) lineage spread from an eastern region of North America towards the north and west. It is possible that the Q-NWT01 (xM265) lineage originated in individuals who later developed into the Dorset culture. The detection of Y-HGs of possible Dorset origin in the current Inuit populations would indicate that there was interbreeding between the individuals of the Dorset and Thule cultures. In light of this hypothesis, a back migration of the Q-NWT01 (xM265) lineage and/or its derived lineages into Asia needs to be considered as the Q-M120 lineage (see Figure A in S1 File) is wide spread at low frequencies in Asian populations [35–37].

Recently, Raghavan et al [38] reported on the mtDNA sequencing and low-coverage whole genome sequencing of archeological human specimens assigned to different Paleo- and Neo-Eskimo cultures. All of the Paleo-Eskimos were found to be a continuum of a single source population [38]. However, gene flow between the Dorset Paleo-Eskimos and the Thule Inuit could not be excluded [38]. As suggested above, the mixture of two paternal lineages in the Inuit populations, both in this study and in [22], can indicate past gene flow between populations such as the Dorset and Thule Inuit. Furthermore, whole-genome deep sequencing of hair from a 4,000 year old Saqqaq Paleo-Eskimo showed that the Saqqaq individual belonged to Y-HG Q1a*-MEH2 (xM120) [39]. Theoretically, the Saqqaq individual could be a carrier of the derived allele at the NWT01 locus, but unfortunately the NWT01 locus was not characterized. Nevertheless, results using mtDNA and autosomal analyses can indeed be different from Y-chromosomal analyses as it is possible that a male-biased gene flow occurred. Deeper genetic analyses of more Inuit populations, from various geographic locations, are needed to characterize the population dynamics of ancient and current Inuit populations in the North Circumpolar Region. However, caution should be taken when interpreting the origin and migration routes of human populations using only Y-chromosomal data as the Y chromosome is very sensitive to drift effects such as founding events and population size fluctuations.

Overall, our results are in agreement with previous studies, indicating that the populations of the North Circumpolar Region, including Greenland, share a common origin with Native American populations. However, the male Inuit population in Greenland appears to be a mixture of individuals belonging to two Y-chromosomal lineages within Y-HG Q-M242. Males carrying the derived state at the M3 locus appear to stem from an expansion of humans from the northwest region of North America. Most likely, these individuals are descendants of the Thule culture that reached Greenland approximately 800 y. a. Individuals carrying the derived state at the NWT01 locus may have been part of the same expansion [22]. However, our results indicate that the Inuit individuals of the Q-NWT01 (xM265) lineage may be descendants of the Dorset Paleo-Eskimo culture. Therefore, our results points to the possibility that there has been gene flow between the Dorset and Thule Inuit and that the current male Inuit population, at least in Greenland, bears traces of both Thule and Dorset descent.

## Materials and Methods

### Samples

A total of 468 randomly sampled male individuals were analyzed, 227 from Greenland and 241 from Denmark. The individuals from Greenland were confirmed to have a birthplace in Greenland. All of the samples involved in the study were anonymized DNA extracts from unrelated male individuals obtained from the biobank of the Department of Forensic Medicine, Copenhagen Denmark (approved by the Danish Data Protection Agency, 2002-54-1080). The use of the samples are in accordance with the Danish Law, LOV nr 593 af 14/06/2011 (Lov om videnskabsetisk behandling af sundhedsvidenskabelige forskningsprojekter (https://www.retsinformation.dk/Forms/R0710.aspx?id=137674), see the Supplemental materials and methods in S1 File), and was approved by the Danish ethical committee (KF-01-037/03, H-1-2011-081 and H3-2012-023). The study complies with the ethical principles of the 2000 Helsinki Declaration of the 206 World Medical Association (http://www.uma.net/e/policy/b3.htm). The DNA was extracted from 200 μl blood using the Blood QiaAmpDNA Mini kit (Qiagen, Stockach Germany) according to manufacturer’s protocol. The DNA was eluted in 50 μl of AE buffer.

### Y-SNP and Y-STR typing

Five multiplexes (Iplex, Qplex, Q1a2Plex, R1aPlexs, and R1bPlex) were constructed using the Assay Design software for the Sequenom MassARRAY system (Sequenom, Hamburg Germany) S3 Table. The 73 Y-SNPs in the five multiplexes defined 67 Y-HGs within Y-HG I-M170, Q-M242, and R-P224, M207. The Y-SNPs were amplified using the iPLEXGold kit (Sequenom, Hamburg Germany) (Table G in S1 File). All of the samples were typed in duplicate. The single base extension (SBE) products were detected using the Sequenom MassARRAY System (Sequenom, Hamburg Germany) according to the manufacturer’s protocol. The Greenlandic individuals were typed hierarchically starting with the Qplex (see the Supplemental materials and methods and Figure C in S1 File). Of the Danish individuals, 171 were previously characterized using Y-SNPs [13], and these individuals were typed with a single multiplex. The Danish individuals who were not previously typed using Y-SNPs were typed hierarchically starting with the R1bPlex (see the Supplemental materials and methods in S1 File). The PCR and SBE primers were purchased from DNA Technology (Risskov Denmark).

The mass spectra were viewed and analyzed in Typer 4.0 (Sequenom, Hamburg Germany). The raw data were further analyzed using the open software R v. 2.15 (http://www.r-project.org/) as described previously [40]. The alleles were called if the peak height was above one. Modifications to the analysis were made for twelve Y-SNPs after inspection of the spectra (Table H in S1 File). Two SNPs, rs3910 (Q1a2Plex) and L584 (R1bPlex), were excluded from the analyses (Table H in S1 File).

All of the Greenlandic individuals were analyzed using the AmpF*l*STRYfiler (Thermo Fisher Scientific, Waltham, MA USA) according to the manufacturer’s protocols in a reaction volume of 10 μl. The amplification products were separated on an ABI 3130xl Genetic Analyzer (Thermo Fisher Scientific, Waltham, MA USA) and visualized using Genmapper IDX v. 1.4 (Thermo Fisher Scientific, Waltham, MA USA) or a combination of GeneScan v. 3.7 (Thermo Fisher Scientific, Waltham, MA USA) and Genotyper v. 3.7 (Thermo Fisher Scientific, Waltham, MA USA). An analytical cut-off signal of 50 RFU was used. The Y-STR profiles were used to predict (http://www.hprg.com/hapest5/) the Y-HGs of the individuals which could not be assigned a Y-HG based on the five multiplexes used in this study.

The results of the Y-SNP and Y-STR typing were submitted to the Y chromosomal haplotype reference database (YHRD), accession number YA003999, YA004000, YA004001, YA004002, YA004003 and YC000109.

### Data analysis

The Greenlandic individuals were divided into groups based on their place of birth according to the four municipalities of Greenland (Figure B in S1 File). The municipality of Sermersooq was further divided into West Sermersooq (Nuuk and Paamiut) and East Sermersooq (Ammassalik and Ittoqqortoormiit).

The Y-SNP haplogroup frequencies were calculated by mere counting. The haplotype diversity, mean number of pairwise differences (MPD) and pairwise genetic distances (*R*_ST_ and *F*_ST_, 10,000 permutations in the Markov chain) were estimated using Arlequin v. 3.5 (http://cmpg.unibe.ch/software/arlequin35/) [41]. The references for the comparative data are presented in Table F in S1 File. The pairwise genetic distances were also calculated using the discrete Laplace method [20] (see the Supplemental materials and methods in S1 File). Pairwise genetic distances were visualized by MDS and plotted in R v. 2.15 using the cmdscale function. The intra-population variances (*Vp*) were calculated as described previously [42]. In short, for each locus the sum of the squared differences between each allele and the mean allele for the locus was divided by the number of individuals minus one.

The median joining networks of the haplotypes (15 Y-STRs) were constructed using Network v. 4.6.1.1 (http://www.fluxus-engineering.com/) [23] and the ρ-statistics were estimated. Weights (1–5) were given to the loci based on the inverse variance of the Y-STRs. The TMRCA was estimated in Network v. 4.6.1.1 [23] using the evolutionary mutation rate [43]. The founder haplotypes were defined as previously described [44]. The TMRCAs for the two Q-M242 lineages were also estimated (15 Y-STRs) using the Bayesian approach implemented in Batwing [45] assuming a population of constant size expanding at time β and priors according to Xue et al [46].

For all of the statistical analyses, DYS385a/b was excluded, because the physical order of the two alleles cannot be determined. Furthermore, DYS389b was calculated by subtraction of the repeat number of DYS389I from the repeat number of DYS389II. In addition, a single haplotype was excluded due to a duplication event in the Y GATA H4 locus.

## Supporting Information

S1 File Supporting methods, figures, and tables. Figure A, A simplified tree of the Q-M242 lineage and the sub-lineages in Q-M242 discussed in this study. Figure B, The distribution of the Y-HGs (Inuit, European and other) in the five investigated regions of Greenland. Inuit: Q-NWT01 (xM265), Q-M3 (xM19, M194, L663, SA01 and L766). European: I-M170, R1a-M513 and R1b-M343. Figure C, A simplified Y-chromosomal tree, including information on the investigation strategy of the five multiplexes. Table A, The diversity of the Y-chromosomal haplogroups in Greenlanders and within the sub-populations in Greenland. Table B, The genetic distances, pairwise *F*_ST_ values below the diagonal and the corresponding p-values above the diagonal based on the Y-chromosomal haplogroup frequencies between five sub-populations in Greenland. Table C, The diversity of the Y-chromosomal haplotypes in Greenlanders and within the sub-populations in Greenland. Table D, The genetic distances, pairwise *R*_ST_ values below the diagonal and the corresponding p-values above the diagonal based on the Y-chromosomal haplotype frequencies, between the five sub-populations in Greenland. Table E, The genetic distances obtained using the discrete Laplace method below the diagonal and the corresponding p-values above the diagonal based on the Y-chromosomal haplotype frequencies between the five sub-populations in Greenland. Table F, The references for the population data used for the comparisons. Table G, The PCR conditions for the five multiplexes. Table H, The SNPs for which modifications were made for analysis in R.
(DOCX)

S1 Table Y-SNPs typed and the number and frequency of each sub-Y-HG covered by the five multiplexes. (XLSX)

S2 Table Y-chromosomal single nucleotide polymorphisms (Y-SNPs) and short tandem repeats (Y-STRs) in Greenlanders and Danes. For Y-SNPs, haplogroups according to International Society of Genetic Genealogy (ISOGG) and mutations in parentheses.
(XLSX)

S3 Table Y-SNPs included in the five multiplexes developed, PCR and SBE primers used. (XLSX)
