# Supplementary material for: Peopling of the North Circumpolar Region – Insights from Y Chromosome STR and SNP Typing of Greenlanders
Source: PLoS One. 2015 Jan 30;10(1):e0116573. doi: 10.1371/journal.pone.0116573 (PMC4312058; doi:10.1371/journal.pone.0116573)
Supplement: S1 File — Figure A, A simplified tree of the Q-M242 lineage and the sub-lineages in Q-M242 discussed in this study. Figure B, The distribution of the Y-HGs (Inuit, European and other) in the five investigated regions of Greenland. Inuit: Q-NWT01 (xM265), Q-M3 (xM19, M194, L663, SA01 and L766). European: I-M170, R1a-M513 and R1b-M343. Figure C, A simplified Y-chromosomal tree, including information on the investigation strategy of the five multiplexes. Table A, The diversity of the Y-chromosomal haplogroups in Greenlanders and within the sub-populations in Greenland. Table B, The genetic distances, pairwise F ST values below the diagonal and the corresponding p-values above the diagonal based on the Y-chromosomal haplogroup frequencies between five sub-populations in Greenland. Table C, The diversity of the Y-chromosomal haplotypes in Greenlanders and within the sub-populations in Greenland. Table D, The genetic distances, pairwise R ST values below the diagonal and the corresponding p-values above the diagonal based on the Y-chromosomal haplotype frequencies, between the five sub-populations in Greenland. Table E, The genetic distances obtained using the discrete Laplace method below the diagonal and the corresponding p-values above the diagonal based on the Y-chromosomal haplotype frequencies between the five sub-populations in Greenland. Table F, The references for the population data used for the comparisons. Table G, The PCR conditions for the five multiplexes. Table H, The SNPs for which modifications were made for analysis in R. (DOCX) [file pone.0116573.s001.docx]

**Supplemental material and methods**

All of the samples involved in the study were anonymized DNA extracts from unrelated male individuals obtained from the biobank of the Department of Forensic Medicine, Copenhagen Denmark (approved by the Danish Data Protection Agency, 2002-54-1080). The use of the samples is in accordance with the Danish Law, LOV nr 593 af 14/06/2011 (Lov om videnskabsetisk behandling af sundhedsvidenskabelige forskningsprojekter (https://www.retsinformation.dk/Forms/R0710.aspx?id=137674)), and was approved by the Danish ethical committee (KF-01-037/03, H-1-2011-081 and H3-2012-023). The study complies with the ethical principles of the 2000 Helsinki Declaration of the 206 World Medical Association (http://www.uma.net/e/policy/b3.htm). Please see the relevant Danish law, LOV nr 593 af 14/06/2011, below with the relevant section underlined.

§ 14. Ethvert sundhedsvidenskabeligt forskningsprojekt skal anmeldes til det videnskabsetiske komitésystem, jf. dog stk. 2-5.

Stk. 2. Spørgeskemaundersøgelser og sundhedsvidenskabelige registerforskningsprojekter skal kun anmeldes til det videnskabsetiske komitésystem, såfremt projektet omfatter menneskeligt biologisk materiale.

Stk. 3. Sundhedsvidenskabelige forskningsprojekter, hvori der alene indgår anonymt menneskeligt biologisk materiale, der er indsamlet i overensstemmelse med lovgivningen på indsamlingsstedet, skal kun anmeldes til det videnskabsetiske komitésystem, hvis forskningsprojektet reguleres i § 25 i lov om kunstig befrugtning i forbindelse med lægelig behandling, diagnostik og forskning m.v.

Google translation:

§ 14 Any health science research must be reported to the scientific ethical committee system, cf. 2-5.

Stk. 2 Research using questionnaire surveys and health records must be reported to the research ethics committee system if the project involves human biological material.

Stk. 3 Health research projects, that include only anonymous human biological materials collected in accordance with the law at the collection site should notify the ethics committee system only if the research is regulated in § 25 of the law on artificial insemination in connection with medical treatment, diagnostics and research, etc.'

All of the Greenlanders were initially typed with the Qplex. Individuals belonging to Y-HG Q1a-L56, L57 (xM19, L529 and M323) were further typed with the Q1a2Plex. Individuals belonging to Y-HG R-P224, M207 were typed with the R1bPlex. Individuals not belonging to Y-HG Q-M242 or R-P224, M207 were typed with the Iplex. Individuals defined as Y-HG R1-M173 were typed with the R1aPlex. Furthermore, individuals not belonging to Y-HG Q-M242, R-P224, M207 or I-M170 were typed with all five multiplexes.

A total of 171 Danes were previously typed for Y-SNPs [[1](#_ENREF_1)]. These individuals were typed with a single multiplex (Iplex, R1aPlex or R1bPlex). The samples that were not previously evaluated using Y-SNPs were typed hierarchically starting with the R1bPlex. Individuals defined as R1-M173 were typed with the R1aPlex. Individuals not belonging to Y-HG R-P224, M207 were typed with the Iplex. Individuals not belonging to Y-HG I-M170 or R-P224, M207 were typed with all of the multiplexes, except Q1a2Plex. See Figure C for a simplified diagram of the location of the five multiplexes in the Y-chromosomal tree.

The discrete Laplace method described by Andersen et al [[2](#_ENREF_2)], can be used to estimate the optimal number (based on Bayesian information criteria) of clusters of individuals based on the Y-STR data [[3](#_ENREF_3)]. Furthermore, the clusters can be used to calculate pairwise genetic distances between sub-populations. In short, the probability of each individual belonging to the respective cluster is estimated. Subsequently, the average probability of a defined set of individuals belonging to a cluster can be calculated. A set of individuals can, for example, be a sub-population defined by geographic origin. The genetic distances between the five defined sub-populations in Greenland were estimated using the discrete Laplace method and visualized using MDS.

**Supplemental figures**


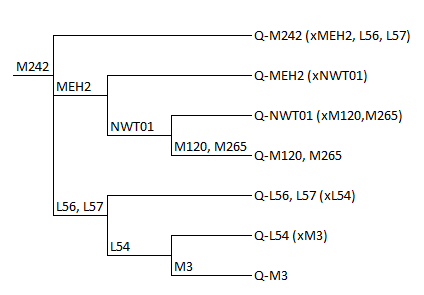


Figure A. A simplified tree of the Q-M242 lineage and the sub-lineages in Q-M242 discussed in this study.


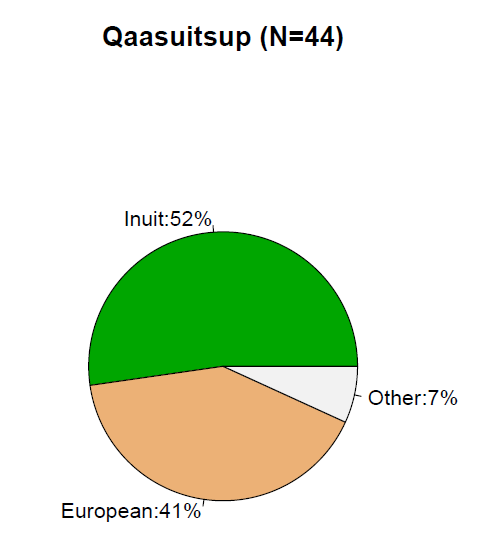

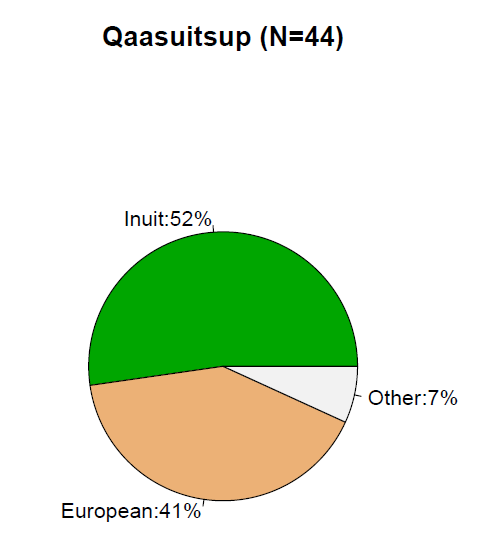

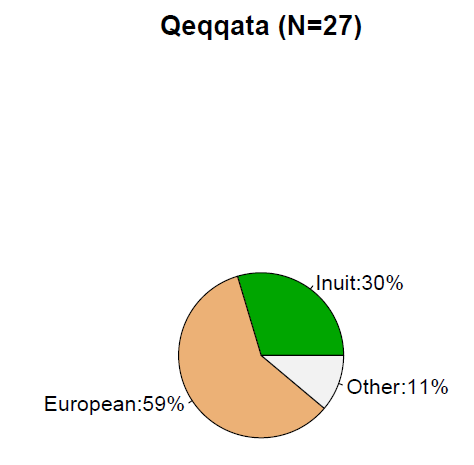

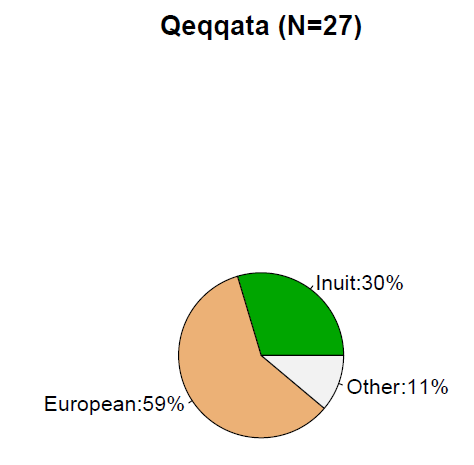

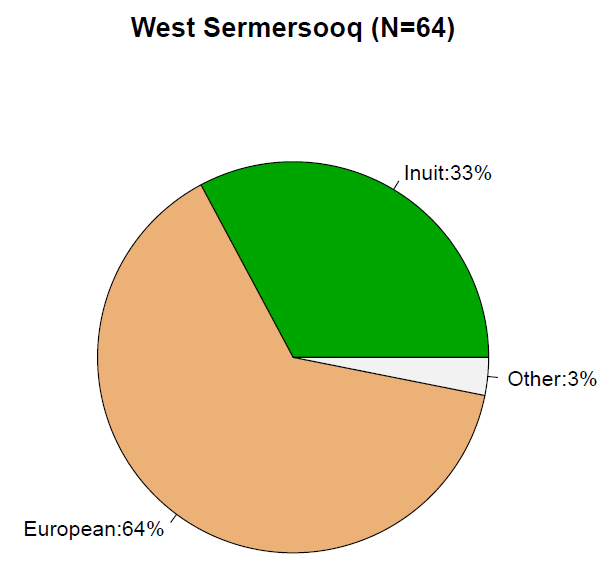

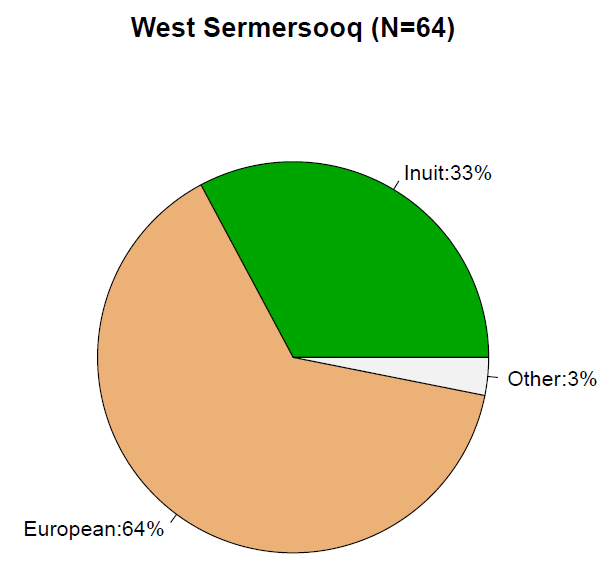

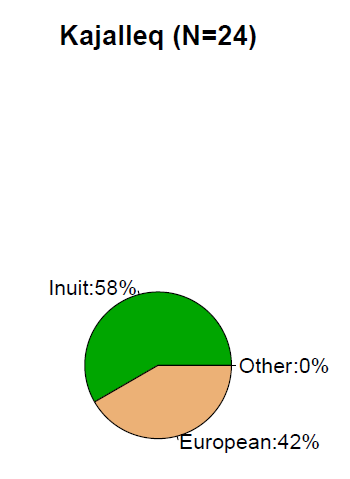

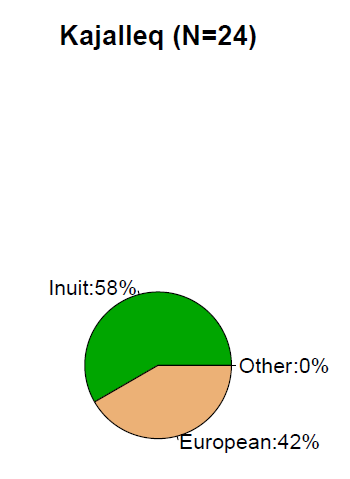

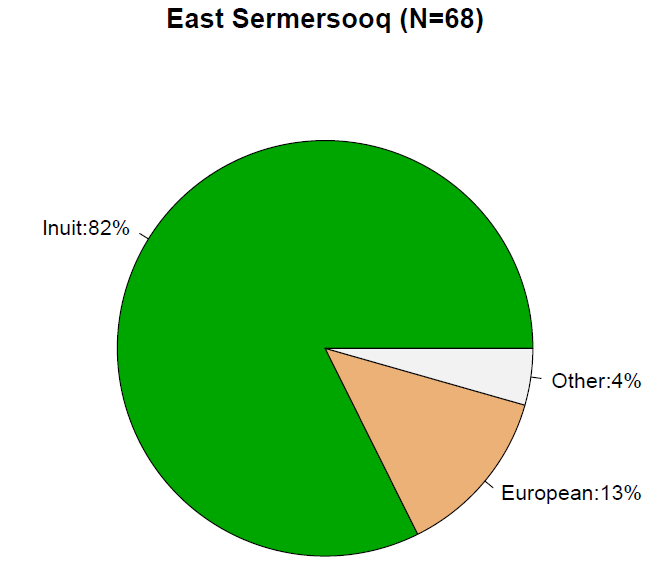

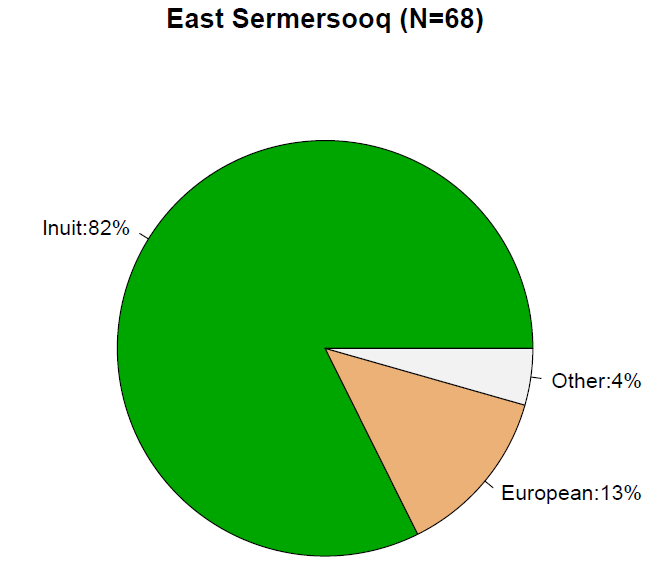

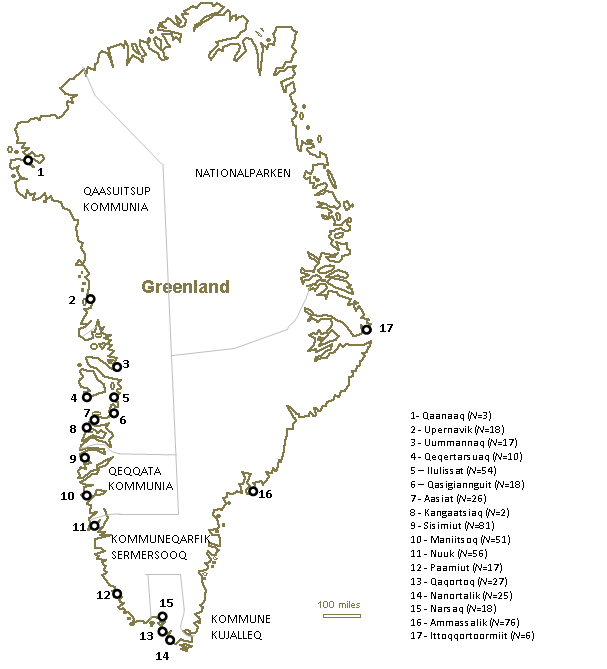


**Qaasuitsup (N=44)**

1. Qaanaaq (*N*=8)

2. Upernavik (*N*=7)

3. Uummannaq (*N*=4)

4. Qeqertarsuaq (*N*=1)

5. Ilulissat (*N*=6)

6. Qasigiannguit (*N*=1)

7. Aasiat (*N*=15)

8.Kangaatsiaq (*N*=2)

**Qeqqata (N=27)**

9. Sisimiut (*N*=10)

10. Maniitsoq (*N*=17)

**West Sermersooq (N=64)**

11. Nuuk (*N*=47)

12. Paamiut (*N*=17)

**Kujalleq (N=24)**

13. Qaqortoq (*N*=8)

14. Nanortalik (*N*=12)

15. Narsaq (*N*=4)

**East Sermersooq (N=68)**

16. Ammassalik (*N*=64)

17. Ittoqqortoormiit (*N*=4)

Figure B. The distribution of the Y-HGs (Inuit, European and other) in the five investigated regions of Greenland. Inuit: Q-NWT01 (xM265), Q-M3 (xM19, M194, L663, SA01 and L766). European: I-M170, R1a-M513 and R1b-M343.


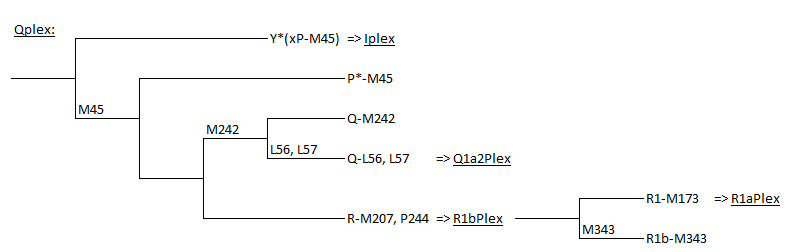


Figure C. A simplified Y-chromosomal tree including information on the investigation strategy of the five multiplexes.

**Supplemental tables**

Table A. The diversity of the Y-chromosomal haplogroups in Greenlanders and within the sub-populations in Greenland.

| **Population** | **N** | **Number of haplogroups** | **Y-chromosomal haplogroup diversity** |
| --- | --- | --- | --- |
| East Sermersooq | 68 | 9 | 0.63 +/- 0.05 |
| Kujalleq | 24 | 7 | 0.79 +/- 0.06 |
| West Sermersooq | 64 | 18 | 0.92 +/- 0.02 |
| Qeqqata | 27 | 11 | 0.91 +/- 0.03 |
| Qaasuitsup | 44 | 13 | 0.85 +/- 0.04 |
| Greenland | 227 | 21 | 0.84 +/- 0.02 |

Table B. The genetic distances, pairwise *F*_ST_ values below the diagonal and the corresponding p-values above the diagonal based on the Y-chromosomal haplogroup frequencies between five sub-populations in Greenland.

|  | **East Sermersooq** | **Kujalleq** | **Qaasuitsup** | **Qeqqata** | **West Sermersooq** |
| --- | --- | --- | --- | --- | --- |
| **East Sermersooq** | * | 0.003+/-0.001 | 0.029+/-0.002 | 0.001+/-0.00005 | 0.000+-0.00005 |
| **Kujalleq** | 0.113 | * | 0.040/+-0.002 | 0.062+/-0.002 | 0.115+-0.003 |
| **Qaasuitsup** | 0.034 | 0.037 | * | 0.209+/-0.003 | 0.123+-0.004 |
| **Qeqqata** | 0.110 | 0.036 | 0.010 | * | 0.707+-0.004 |
| **West Sermersooq** | 0.099 | 0.016 | 0.010 | -0.007 | * |

Table C. The diversity of the Y-chromosomal haplotypes in Greenlanders and within the sub-populations in Greenland.

| **Population** | **N** | **h** | **HT diversity** | **MPD** | **Vp** |
| --- | --- | --- | --- | --- | --- |
| East Sermersooq | 68 | 24 | 0.91 +/- 0.02 | 7.04 +/- 3.35 | 0.63 |
| Kujalleq | 24 | 19 | 0.98 +/- 0.02 | 8.85 +/- 4.23 | 0.72 |
| West Sermersooq | 63* | 55* | 0.99 +/- 0.00 | 9.93 +/- 4.60 | 0.93 |
| Qeqqata | 27 | 23 | 0.98 +/- 0.02 | 9.93 +/- 4.69 | 0.94 |
| Qaasuitsup | 44 | 34 | 0.99 +/- 0.01 | 9.19 +/- 4.31 | 0.94 |
| Greenland | 226* | 120* | 0.98 +/- 0.0005 | 9.20 +/- 4.25 | 0.88 |

* One haplotype excluded due to duplication of Y GATA H4.

Table D. The genetic distances, pairwise *R*_ST_ values below the diagonal and the corresponding p-values above the diagonal based on the Y-chromosomal haplotype frequencies, between the five sub-populations in Greenland.

|  | **East Sermersooq** | **Kujalleq** | **Qaasuitsup** | **Qeqqata** | **West Sermersooq** |
| --- | --- | --- | --- | --- | --- |
| **East Sermersooq** | * | 0.000+/-0.00005 | 0.00+/-0.00005 | 0.000+/-0.00005 | 0.000+/-0.00005 |
| **Kujalleq** | 0.133 | * | 0.150+/-0.003 | 0.131+/-0.003 | 0.160+/-0.004 |
| **Qaasuitsup** | 0.093 | 0.019 | * | 0.266+/-0.004 | 0.070+/-0.003 |
| **Qeqqata** | 0.169 | 0.024 | 0.007 | * | 0.882+/-0.004 |
| **West Sermersooq** | 0.176 | 0.015 | 0.020 | -0.015 | * |

Table E. The genetic distances obtained using the discrete Laplace method below the diagonal and the corresponding p-values above the diagonal based on the Y-chromosomal haplotype frequencies between the five sub-populations in Greenland.

|  | **East Sermersooq** | **Kujalleq** | **Qaasuitsup** | **Qeqqata** | **West Sermersooq** |
| --- | --- | --- | --- | --- | --- |
| **East Sermersooq** | * | 0.006 | 0.004 | 0.001 | 0.00005 |
| **Kujalleq** | 0.115 | * | 0.115 | 0.376 | 0.348 |
| **Qaasuitsup** | 0.106 | 0.093 | * | 0.259 | 0.074 |
| **Qeqqata** | 0.188 | 0.070 | 0.064 | * | 0.959 |
| **West Sermersooq** | 0.217 | 0.054 | 0.063 | 0.012 | * |

Table F. The references for the population data used for the comparisons.

| **Populations** | **Geographic Region** | **Reference** |
| --- | --- | --- |
| Gwich'in | Canadian Northwest Territories | Dulik et al.[[4](#_ENREF_4)] |
| Tlicho | Canadian Northwest Territories | Dulik et al.[[4](#_ENREF_4)] |
| Inuvialuit | Canadian Northwest Territories | Dulik et al.[[4](#_ENREF_4)] |
| Inupiat | Alaska | Davis et al.[[5](#_ENREF_5)] |
| Yupik | Alaska | Davis et al.[[5](#_ENREF_5)] |
| Athabaskan | Alaska | Davis et al.[[5](#_ENREF_5)] |
| Tingit  Haida | Southeast Alaska | Schurr et al.[[6](#_ENREF_6)] |
| Altai-kizhi  Chelkans  Kumandins  Tubalars | Southern Siberia | Dulik et al.[[7](#_ENREF_7)] |
| Multi-ethnical | North Bolivia | Tirado et al.[[8](#_ENREF_8)] |
| Rio | Brazil | Sanchez-Diz et al.[[9](#_ENREF_9)] |
|  | Brazil | Sanchez-Diz et al.[[9](#_ENREF_9)] |
| Buenos Aries | Argentina | Sanchez-Diz et al.[[9](#_ENREF_9)] |
| Tamang  Newar  Kathmandu | Nepal | Gayden et al.[[10](#_ENREF_10)] |
| Newar | Nepal | Gayden et al.[[10](#_ENREF_10)] |
| Han Chinese | Shanxi Province, Northern China | Bai et al.[[11](#_ENREF_11)] |
|  | South Korea | Kim et al.[[12](#_ENREF_12)] |
|  | Portugal | Sanchez-Diz et al.[[9](#_ENREF_9)] |
|  | Denmark | This study |
|  | Italy | Turrina et al.[[13](#_ENREF_13)] |

Table G. The PCR conditions for the five multiplexes.

| 94°C | 2 min |  | MgCl_2_ concentration:  Iplex: 2 mM  Qplex: 2 mM  Q1a2Plex: 4 mM  R1aPlex: 3 mM  R1bPlex: 2 mM |
| --- | --- | --- | --- |
| 94°C | 20 sec | x45 |  |
| X°C  Iplex: 56°C  Qplex: 62°C  Q1a2Plex: 58°C  R1aPlex: 62°C  R1bPlex: 60°C | 30 sec |  |  |
| 72°C | 1 min |  |  |
| 72°C | 3 min |  |  |
| 4°C | Hold |  |  |

Table H. The SNPs for which modifications were made for analysis in R.

| **SNP** | **Plex** | **Modification** |
| --- | --- | --- |
| S337 | Iplex | AB for homozygous allele calls were allowed to be 0.7 |
| rs17307294 | Iplex | AB for homozygous allele calls were allowed to be 0.7 |
| L596 | Iplex | AB for homozygous allele calls were allowed to be 0.7 |
| L232 | Qplex | AB for homozygous allele calls were allowed to be 0.7 |
| L804 | Q1a2Plex | AB for homozygous allele calls were allowed to be 0.7 |
| SA01 | Q1aPlex | AB for homozygous allele calls were allowed to be 0.7 |
| rs2032677 | Q1a2Plex | AB for homozygous allele calls were allowed to be 0.7 |
| rs3910 | Q1a2Plex | Excluded due to extra peak around true calls |
| M458 | R1aPlex | AB for homozygous allele calls were allowed to be 0.7 |
| S339 | R1aPlex | AB for homozygous allele calls were allowed to be 0.7 |
| rs2032658 | R1aPlex | AB for homozygous allele calls were allowed to be 0.7 |
| L217-1 | R1bPlex | AB for homozygous allele calls were allowed to be 0.7 |
| rs9786184 | R1bPlex | AB for homozygous allele calls were allowed to be 0.7 |
| L584 | R1bPlex | Excluded due to calls not corresponding to information in database |

AB: Allele balance calculated as (height of allele 1 - height of allele 2)/(height of allele 1 + height of allele 2)

**Supplemental references**

1. Sanchez JJ, Børsting C, Hernandez A, Mengel-Jørgensen J, Morling N (2004) Y chromosome SNP haplogroups in Danes, Greenlanders and Somalis. International Congress Series 1261: 347-349.

2. Andersen MM, Eriksen PS, Morling N (2013) The discrete Laplace exponential family and estimation of Y-STR haplotype frequencies. J Theor Biol 329: 39-51.

3. Andersen MM, Eriksen PS, Morling N (2014) Cluster analysis of European Y-chromosomal STR haplotypes using the discrete Laplace method. Forensic Sci Int Genet 11: 182-194.

4. Dulik MC, Owings AC, Gaieski JB, Vilar MG, Andre A, et al. (2012) Y-chromosome analysis reveals genetic divergence and new founding native lineages in Athapaskan- and Eskimoan-speaking populations. Proc Natl Acad Sci U S A 109: 8471-8476.

5. Davis C, Ge J, Chidambaram A, King J, Turnbough M, et al. (2011) Y-STR loci diversity in native Alaskan populations. Int J Legal Med 125: 559-563.

6. Schurr TG, Dulik MC, Owings AC, Zhadanov SI, Gaieski JB, et al. (2012) Clan, language, and migration history has shaped genetic diversity in Haida and Tlingit populations from Southeast Alaska. Am J Phys Anthropol 148: 422-435.

7. Dulik MC, Zhadanov SI, Osipova LP, Askapuli A, Gau L, et al. (2012) Mitochondrial DNA and Y chromosome variation provides evidence for a recent common ancestry between Native Americans and Indigenous Altaians. Am J Hum Genet 90: 229-246.

8. Tirado M, Lopez-Parra AM, Baeza C, Bert F, Corella A, et al. (2009) Y-chromosome haplotypes defined by 17 STRs included in AmpFlSTR Yfiler PCR Amplification Kit in a multi ethnical population from El Beni Department (North Bolivia). Leg Med (Tokyo) 11: 101-103.

9. Sanchez-Diz P, Alves C, Carvalho E, Carvalho M, Espinheira R, et al. (2008) Population and segregation data on 17 Y-STRs: results of a GEP-ISFG collaborative study. Int J Legal Med 122: 529-533.

10. Gayden T, Chennakrishnaiah S, La Salvia J, Jimenez S, Regueiro M, et al. (2011) Y-STR diversity in the Himalayas. Int J Legal Med 125: 367-375.

11. Bai R, Zhang Z, Liang Q, Lu D, Yuan L, et al. (2013) Haplotype diversity of 17 Y-STR loci in a Chinese Han population sample from Shanxi Province, Northern China. Forensic Sci Int Genet 7: 214-216.

12. Kim SH, Han MS, Kim W (2010) Y chromosome homogeneity in the Korean population. Int J Legal Med 124: 653-657.

13. Turrina S, Atzei R, De Leo D (2006) Y-chromosomal STR haplotypes in a Northeast Italian population sample using 17plex loci PCR assay. Int J Legal Med 120: 56-59.
